# Clinical characteristics and outcome of patients with enterococcal liver abscess

**DOI:** 10.1038/s41598-021-01620-9

**Published:** 2021-11-15

**Authors:** K. Große, D. Ohm, S. Würstle, J. F. Brozat, R. M. Schmid, C. Trautwein, A. Stallmach, T. Bruns, Philipp A. Reuken

**Affiliations:** 1 Department of Internal Medicine III, University Hospital RWTH Aachen, Aachen, Germany; 2 Department of Internal Medicine IV, Jena University Hospital, Friedrich Schiller University, Erlanger Allee 110, 07747 Jena, Germany; 3 Department of Internal Medicine II, School of Medicine, University Hospital Rechts der Isar, Technical University of Munich, Munich, Germany

**Keywords:** Liver diseases, Bacterial infection

## Abstract

Epidemiology of bacteria isolated from pyogenic liver abscesses change, and an increase in enterococci has been reported in European hospitals. The aim of this study was to investigate the clinical characteristics and outcome of enterococcal PLA. We performed a retrospective analysis of patients with microbiologically confirmed PLA at three German university centers. Indicators of enterococcal PLA were determined using binary logistic regression, and survival analysis was performed using Kaplan–Meier statistics and Cox regression analysis. Enterococci were isolated in 51/133 (38%) patients with PLA. Patients with enterococcal PLA had smaller abscess diameter (4.8 vs. 6.7 cm, *p* = 0.03) than patients with non-enterococcal PLA, but had more frequent polymicrobial culture results. In univariate logistic regression analysis, alcohol abuse (OR 3.94, 95% CI 1.24–12.49, *p* = 0.02), hepatobiliary malignancies (OR 3.90, 95% CI 1.86–8.18, *p* < 0.001) and cirrhosis (OR 6.36, 95% CI 1.27–31.96, *p* = 0.02) were associated with enterococcal PLA. Patients with enterococcal PLA had a higher mortality than patients with non-enterococcal PLA (hazard ratio 2.92; 95% confidence interval 1.09–7.80; *p* = 0.03), which remained elevated even after excluding patients with hepatobiliary malignancies, cirrhosis, and transplant recipients in a sensitivity analysis. The increased mortality was associated with non-fecal enterococci but not in patients with *Enterococcus faecalis*. In this retrospective, multicenter study, enterococcal PLA was common and indicated an increased risk of mortality, underscoring the need for close clinical monitoring and appropriate treatment protocols in these patients.

## Introduction

Pyogenic liver abscess (PLA) is an important diagnosis in patients with fever of unknown cause. In Western countries, the incidence ranges from 1.0 to 3.6 per 100,000^1^, but in Asia it can reach up to 17 per 100,000^2^ and the mortality rate is up to 15%^3^. Treatment of a PLA is based on empiric antibiotic therapy in combination with percutaneous drainage, either ultrasound- or CT-guided, or surgical resection for multilocular abscesses. Antibiotic therapy is based on third-generation cephalosporins (TGC) or Piperacillin/Tazobactam (PTZ)^4^. However, knowledge of the microbial spectrum and resistance profiles is critical for choosing the most effective empirical treatment.

The vast majority of PLA are caused by bacterial pathogens, but there are notable differences between different regions. Studies from Southeast Asia report a high proportion of PLA caused by *Klebsiella pneumoniae*, whereas in Western countries Gram-negative Enterobacterales such as *Escherichia coli* as well as Gram-positive cocci are the most common pathogens^1,2,5–8^ with increasing prevalence of enterococci and multidrug-resistant bacteria^9–11^.

Among Gram-positive isolates, enterococci are of great clinical importance because they are naturally resistant to TGC and, in the case of *Enterococcus faecium*, often exhibit beta-lactam resistance. Enterococci are frequently detected in intra-abdominal infections such as cholangitis, spontaneous bacterial peritonitis, or infected pancreatic necrosis^12–15^. A recent study from a German tertiary center^9^ reported that enterococci were the most common pathogen in PLA (29%), with one in three enterococci identified being resistant to vancomycin. However, the impact of enterococcal infections on mortality is controversial^16,17^, and enterococci have also been associated with mortality in intra-abdominal infections in some studies^12^.

Therefore, the aim of this retrospective cross-sectional study was to investigate the bacterial spectrum and antibacterial resistance profile in patients with PLA from three German centers and to analyze the association of enterococcal PLA with survival.

## Patients and methods

### Study design

To characterize the pathogen spectrum and resistance patterns in patients with PLA, data from patients who underwent microbiological testing between 2009 and January 2020 were retrospectively collected at three German university centers (Jena University Hospital, Aachen University Hospital, and Technical University of Munich). Microbiological sampling from the PLA was performed either by percutaneous puncture guided by ultrasound or computed tomography (CT), or during surgical procedures. Microbial cultures were processed and analyzed according to standard local procedures. Patients were on antibiotics prior to microbiological samplings (n = 47, 35%) or received antibiotics directly after puncture (n = 86, 65%). Medical records, including patient records, electronic health records, imaging data, laboratory data, and nursing documentation were used to retrospectively determine clinical and laboratory data. The following variables were recorded in the medical records: Age, sex, comorbidities, medication, including antibiotics and changes in antibiotic therapy, intensive care unit (ICU) treatment, endoscopic diagnostic procedures, and interventions, length of hospital stay, and mortality. Patients without microbiological growth or without calculated antimicrobial therapy were excluded from the analysis. The study protocol conformed to the ethical guidelines of the 1975 Declaration of Helsinki and was approved by the internal review boards (University Hospital Jena 3783-05/13 University of Munich 614/19 S-KH and University Hospital Aachen EK125-20) and written informed consent was waived by the ethics committees of the Jena University Hospital, the ethics committee of Aachen University Hospital and the ethics committee of Technical University Munich as only routine data was used.

Gram-negative bacteria were defined as multidrug-resistant if they were non-susceptible to at least one out of three antimicrobial categories according to the interim definition of the European Centre for Disease Prevention and Control (ECDC) and the Centers for Disease Control and Prevention (CDC)^18^. In addition, methicillin-resistant *Staphylococcus aureus* (MRSA) and vancomycin-resistant *Enterococcus* spp. (VRE) were defined as multidrug-resistant bacteria.

### Statistical analysis

Categorical variables were expressed as absolute and relative frequencies and compared using Fisher's exact test. Continuous variables were expressed as medians with interquartiles and compared using the Mann–Whitney U test as variables were not normally distributed. Indicators of enterococcal liver abscess were determined by univariate and multivariable binary logistic regression analysis. Survival analysis was performed using Kaplan–Meier statistics and Cox regression analysis. Survival data were right-censored at loss to follow-up or at 365 days, whichever occurred earlier. Multivariable Cox proportional hazards models were used to evaluate the effects of covariates on short-term survival. A significance level of 0.05 was selected for all statistical tests. Analyses were performed using IBM SPSS Statistics 27 (IBM Corp., Armonk, NY, USA), and data were plotted using Prism 8 (GraphPad, La Jolla, CA, USA).

## Results

### Baseline characteristics

**Figure 1:**
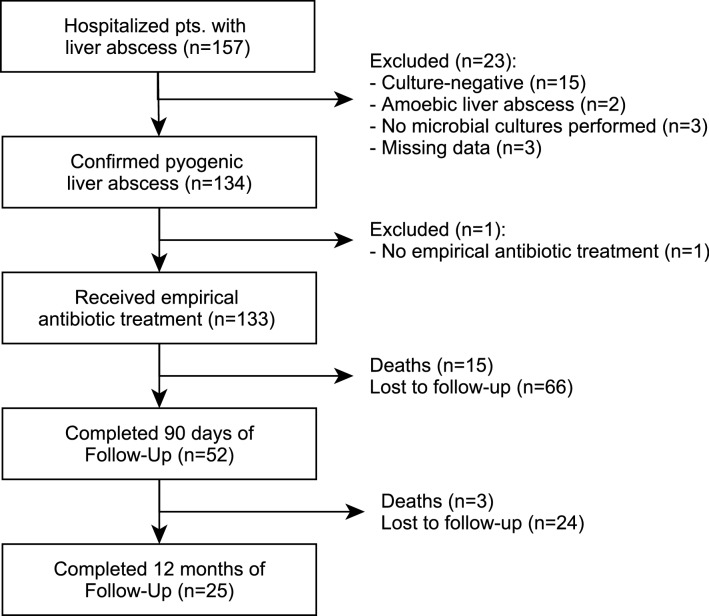
Patients included into the analysis.

A total of 157 patients with liver abscess were identified at the three study centers. Twenty-four patients were excluded from the analysis because of negative cultures (n = 15; 9.5%), amebic abscess (n = 2; 1.2%), no microbiological sampling (n = 3; 1.9%), no empirical treatment (n = 1; 0.6%), or other missing data (n = 3; 1.9%), and 133 patients empirically treated for PLA were included in the analysis (Fig. 1).

The median age of the patients was 65 years and 79 patients (59%) were male. Fifty-two patients (39%) had a history of hepatobiliary carcinoma, and 60 patients (48%) had previously undergone biliary stenting. The median abscess diameter was 5.6 cm. Abscess drainage was performed in 87% of patients. For empiric therapy, the most commonly used antibiotic was piperacillin/tazobactam in 42 patients (32%), followed by ampicillin/sulbactam (23 patients; 17%), fluoroquinolones (22 patients; 16%), carpabenems (20 patients; 15%), and ceftriaxone (19 patients; 14%). Twenty-nine patients (22%) received combination therapy with metronidazole, vancomycin was added in 6 patients (5%), and linezolid was added in 2 patients (2%). Empirical therapy did not correlate with microbial spectrum or patient outcome (data not shown).

The majority of culture results were polymicrobial (72 cultures; 54%) with a median number of two bacterial species. The most frequently isolated pathogens were from the order Enterobacterales in 92 patients (69%), followed by enterococci (51 patients, 38%) and streptococcal species (25 patients, 19%) (Table 1). Most frequent Enterobacterales were *E. coli (*60 patients; 45%), *Klebsiella* spp. (30 patients,;23%) and *Citrobacter* spp. (13 patients; 10%).

**Table 1:** Baseline characteristics, microbial spectrum and treatment in patients with pyogenic liver abscess stratified by outcome.

	Missing data	Patients with PLA (N = 133)	Survivors (N = 115)	Non-survivors (N = 18)	*P* value
**Baseline characteristics**					
Age (years)	0	65 (56–74)	64 (55–74)	65 (58–74)	0.49
Male sex	0	79 (59)	47 (41)	7 (39)	1.00
Smoker	19	32 (28)	27 (28)	5 (31)	0.77
Alcohol use disorder	23	15 (14)	12 (13)	3 (19)	0.46
Hepatobiliary cancer (incl. liver metastasis and pancreatic cancer)	0	52 (39)	48 (42)	4 (22)	0.76
Previous bile duct stent	8	60 (48)	49 (45)	11 (65)	0.19
Cirrhosis	0	9 (7)	7 (6)	2 (11)	0.35
Liver transplant recipient	0	10 (8)	6 (5)	4 (22)	0.03*
COPD	1	16 (12)	15 (13)	1 (6)	0.70
Long-term dialysis	1	11 (8)	7 (6)	4 (22)	0.04*
Previous surgery	1	56 (42)	49 (43)	7 (39)	0.80
Antibiotic exposure	1	27 (20)	22 (19)	5 (29)	0.34
**Abscess criteria**					
Abscess diameter (cm)	23	5.6 (3.7–8.1)	5.7 (3.8–8.5)	4.9 (3.1–6.8)	0.16
Polymicrobial culture results	0	72 (54)	63 (55)	9 (50)	0.80
Number of bacterial species	0	2 (1–2)	2 (1–2)	1.5 (1–3)	1.00
Anaerobes	0	20 (15)	19 (17)	1 (6)	0.31
Enterobacterales	0	92 (69)	80 (70)	12 (67)	0.79
Enterococci	0	51 (38)	39 (34)	12 (67)	0.02*
Streptococci	0	25 (19)	25 (22)	0 (0)	0.02*
Non-fermenters	0	7 (5)	6 (5)	1 (6)	1.00
ESBL-Enterobacterales	0	20 (15)	17 (15)	3 (17)	0.73
CR-Enterobacterales	0	2 (2)	2 (2)	0 (0)	1.00
VRE	0	9 (7)	5 (5)	3 (17)	0.10
MRSA/MRSE	0	3 (2)	2 (2)	1 (6)	0.36
**Management**					
Intensive care	0	39 (29)	28 (24)	11 (61)	0.004*
Abscess drainage	1	115 (87)	98 (86)	17 (94)	0.47

Medians with interquartile ranges and frequencies with percentages are shown. *P* values from Mann–Whitney U test or Fisher’s exact test. *p < 0.05.

*PLA* pyogenic liver abscess, *MRSA* methicillin-resistant Staphylococcus aureus, *MRSE* methicillin-resistant Staphylococcus epidermidis, *VRE* Vancomycin-resistant enterococci, *ESBL* extended-spectrum beta-lactamase, *CR* Carbapenem-resistant.

Isolated enterococci included 29 isolates of *E. faecium*, 16 isolates of *Enterococcus faecalis* and 6 other enterococci. The isolation of enterococci did not differ between the three study centers (Aachen 38.6%, Munich 40.9% and Jena 34.3% of the patients).

### Characteristics of patients with enterococcal pyogenic liver abscess

Patients with enterococcal PLA had a smaller abscess diameter (4.8 vs. 6.7 cm, *p* = 0.03) than patients with non-enterococcal PLA but were more likely to have polymicrobial culture results (75% vs. 41%, *p* < 0.001) (Table 2).

**Table 2:** Baseline characteristics, microbial spectrum and treatment in patients with pyogenic liver abscess stratified by the isolation of *Enterococcus spp.*

	Non-enterococcal PLA (n = 82)	Enterococcal PLA (n = 51)	*P* value
Age (years)	65 (53–75)	64 (59–70)	0.71
Male sex	46 (56)	33 (65)	0.37
Smoker	15 (21)	17 (39)	0.06
Alcohol use disorder	5 (7)	10 (24)	0.02
Hepatobiliary cancer (incl. liver metastasis and pancreatic cancer)	22 (27)	30 (59)	< 0.001
Previous bile duct stent	34 (45)	26 (53)	0.46
Cirrhosis	2 (2)	7 (14)	0.03
Liver transplant recipient	6 (7)	4 (8)	1.00
Chronic obstructive pulmonary disease	5 (6)	11 (22)	0.01
Long-term dialysis	2 (2)	9 (18)	0.003
Previous surgery	29 (35)	27 (54)	0.046
Antibiotic exposure	16 (20)	11 (22)	0.83
Polymicrobial abscess culture	34 (41)	38 (75)	< 0.001
Abscess diameter (cm)	6.7 (4–8.8)	4.8 (3.1–7.5)	0.03
Intensive care	17 (21)	22 (42)	0.02
Death within 365 days	6 (7)	12 (24)	0.02

Medians with interquartile ranges and frequencies with percentages are shown. *P* values from Mann–Whitney U test or Fisher’s exact test.

*PLA* Pyogenic liver abscess.

There were no differences in age (64 vs. 65 years, *p* = 0.71) and gender (male 65% vs. 56%, *p* = 0.37) between the two groups. Patients with enterococcal liver abscess were significantly more likely to have cirrhosis (14% vs. 2%, *p* = 0.03) or hepatobiliary malignancies (59% vs. 27%, *p* < 0.001). The number of patients who had previously undergone biliary stenting (53% vs. 45%, *p* = 0.46) or liver transplantation (8% vs. 7%, *p* = 1.00) did not differ between the two groups. Concomitant diseases other than liver were also more common in patients with enterococcal PLA, such as long-term dialysis (18% vs. 2%; *p* = 0.003), chronic obstructive pulmonary disease (COPD) (22% vs. 6%, *p* = 0.01), and previous intra-abdominal surgery (54% vs. 35%, *p* = 0.046). Notably, prior antibiotic exposure within 14 days to sampling was not associated with enterococcal isolation (22% vs. 20%, *p* = 0.83).

In univariate logistic regression analysis, alcohol use disorder (OR 3.94, 95%-CI 1.24–12.49, *p* = 0.02), hepatobiliary malignancies (OR 3.90, 95%-CI 1.86–8.18, *p* < 0.001) and cirrhosis (OR 6.36, 95%-CI 1.27–31.96, *p* = 0.02) were associated with enterococcal PLA. In multivariate analysis with stepwise backward elimination, alcohol abuse (OR 6.16, 95%-CI 1.75–21.68, *p* = 0.005) and hepatobiliary malignancies (OR 5.29, 95%-CI 2.18–12.82, *p* < 0.001) were found to be independent indicators PLA caused by *Enterococcus* spp. (Table 3).

**Table 3:** Indicators of enterococcal pyogenic liver abscess.

	Univariate logistic regression			Multivariable logistic regression*		
	Univariate Odds ratio	95% CI	*P* value	Adjusted Odds ratio	95% CI	*P* value
Alcohol abuse documented	3.94	1.24–12.49	0.02	6.16	1.75–21.68	0.005
Hepatobiliary cancer (incl. liver metastasis and pancreatic cancer)	3.90	1.86–8.18	< 0.001	5.29	2.18–12.82	< 0.001
Cirrhosis	6.36	1.27–31.96	0.02	Excluded from model		

*Backward stepwise conditional exclusion model. CI Confidence interval.

### Survival analysis

Within 12 months, 18 of the 133 patients died, 15 of them within the first 90 days. (Fig. 1). Demographic characteristics did not differ between survivors and non-survivors. Liver transplant recipients (22% vs. 5%; *p* = 0.03) with PLA and patients receiving long-term hemodialysis (22% vs. 6%, *p* = 0.04) were at significantly higher risk for fatal outcome. In terms of underlying pathogen spectrum, non-survivors had a lower prevalence of streptococci (0% vs. 22%; *p* = 0.02) and a higher prevalence of enterococci (67% vs. 34%, *p* = 0.02). There were no significant differences in ESBL-producing Enterobacterales (17% vs. 15%; *p* = 0.79), carbapenem-resistant Enterobacterales (0% vs. 2%; *p* = 1.00), or MRSA/MRSE (6% vs. 2%; *p* = 0.36) between non-survivors and survivors (Table 1).

**Figure 2:**
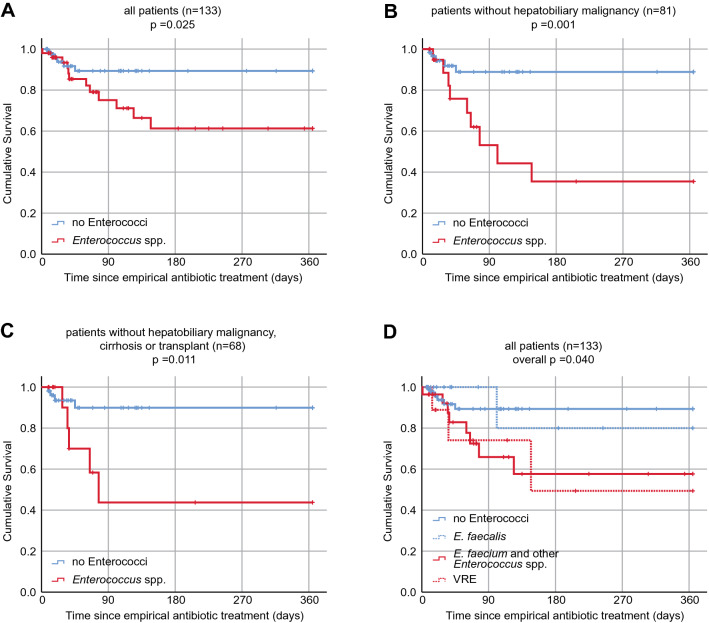
Survival in patients with enterococcal pyogenic liver abscess.

Within 365 days, 12 of the patients with enterococcal PLA died compared to 6 patients with non-enterococcal PLA (*p* = 0.025 by log-rank test) (Fig. 2A). This difference persisted in the sensitivity analysis when only patients without hepatopancreatic malignancy (Fig. 2B) or only patients without hepatopancreatic malignancy, cirrhosis and transplantation (Fig. 2C) were analyzed. The univariate hazard ratio for death was 2.92 (95% CI 1.09–7.80; *p* = 0.03) in patients with enterococcal PLA (Table 4).

**Table 4:** Association of enterococcal pyogenic liver abscess with mortality.

	Univariate Cox regression			Adjusted Cox regression*		
	Univariate hazard ratio	95% CI	*P* value	Adjusted hazard ratio	95% CI	*P* value
Enterococcal pyogenic liver abscess	2.92	1.09–7.80	0.03	2.84	1.06–7.62	0.04
No enterococci	1.00 (ref.)			1.00 (ref.)		
*E. faecalis*	0.93	0.11–7.74	0.95	0.88	0.10–7.44	0.91
*E. faecium* and other enterococci incl. VRE	3.63	1.34–9.83	0.01	3.52	1.29–9.58	0.01

*Adjusted for age and sex.

*CI* Confidence interval, *VRE* Vancomycin-resistant enterococci.

The presence of *E. faecalis* was associated with mortality comparable to that of patients with non-enterococcal PLA (HR 0.93; 95%-CI 0.11–7.72; *p* = 0.95), whereas the presence of *Enterococcus faecium* or non-faecalis non-faecium *Enterococcus* species was associated with significantly higher mortality (HR 3.63, 95%-CI 1.34–9.83, *p* = 0.01) (Fig. 2D, Table 4). The associations of PLA by Enterococcus spp. and *E. faecium* with survival remained significant after adjustment for age and sex (Table 4).

## Discussion

In this retrospective analysis from three German tertiary centers, we demonstrate that enterococci can be isolated in more than one third of patients with PLA and indicate an increased risk of mortality. The high proportion of enterococci in PLA is consistent with another recent study from Central Europe, which shows *Enterococcus* spp. as the most common pathogen in PLA^9^, but significantly higher than in Asian cohorts^2,7,8^.

At present, the most common route of PLA is ascending cholangitis and invasion of liver parenchyma on the ground of obstruction by gallstone disease, strictures or malignancy^19^. Cohorts from our and other European University centers^9^ are often enriched for patients with underlying hepatobiliary strictures who underwent prior biliary tract interventions. In patients with malignant biliary obstruction, antibiotic exposure predisposes to enterococcal bactibilia^20^, presumably predisposing to cholangiogenic liver abscess by enterococci. In our study, 48% of patients had a bile duct stent previously or currently implanted, which can often be colonized with enterococci in addition to Gram-negative bacteria or fungi^14,21,22^. However, in contrast to our previous analysis of acute ascending cholangitis^14,21^, neither the presence of biliary stents nor previous antibiotic exposure was significantly associated with enterococcal PLA in our analysis. In the present study, only alcohol use disorders, cirrhosis, and hepatobiliary cancer were linked to isolation of *Enterococcus* spp. from PLA in univariate analysis. Considering that gut flora reach the biliary system either often by ascent from the intestine, fecal enterococcal overgrowth as seen after TGC use^23^ or changes in the microbiota composition may contribute. The gut microbiota of patients with alcohol use disorders and alcoholic liver disease is often enriched in *Enterococcus* spp, especially when other risk factors such as proton pump inhibitors are present^24,25^.

Enterococci are part of the physiological flora of the gastro-intestinal tract. It remains controversial whether enterococci have pathologic significance, as they are usually described as having low virulence^17^. In our analysis, isolation of enterococci was associated with higher mortality, even after exclusion of patients with concomitant hepatobiliary disease, suggesting a possible influence of enterococci on mortality. In contrast to other intraabdominal infections in vulnerable patients^12^, we did not observe a correlation between coverage of enterococci by empirical therapy and outcome. This could be due to the fact that PLA requires several weeks of antibiotic therapy and the duration of empiric therapy is rather short in relation to definitive therapy.

Over the last years, vancomycin-resistant *Enterococcus* spp. increased, accounting for 19% of all Enterococcus isolates in blood stream infections^26^ and up to 33% of enterococcal isolates from PLA^9^. In our analysis, patients with PLA due to *E. faecalis*, which is usually susceptible to beta-lactams and vancomycin^27^, had a prognosis comparable to that of non-enterococcal PLA, whereas more resistant enterococcal species such as *E. faecium* with and without vancomycin resistance were associated with higher mortality. The main cause of the increasing proportion of VRE is antibiotic exposure. Therefore, from an antibiotic stewardship perspective, our results may justify deferring enterococcal-specific therapy in non-critically ill patients with PLA until culture results are available. However, this needs to be clarified in a larger, prospective, randomized cohort.

This study has some limitations. First, it is a retrospective analysis of complex and often lengthy multimodal therapeutic approaches. In particular, a structured follow-up including data on the resolution of PLA were missing in some cases. Although the phenotypic characteristics of enterococcal PLA are quite robust because only patients with microbiologically confirmed PLA were included, there is a risk of bias in the mortality analysis because approximately 50% of patients were lost to follow-up within 90 days. Second, because of the nature of a noninterventional observational study, we could not distinguish whether enterococci were causative for the increased mortality or merely a surrogate marker for sicker patients. Third, all study sites were university hospitals, so patients with biliary structures, repeated antibiotic exposure, and in an immunocompromised state were likely overrepresented. However, the association between enterococci and increased mortality remained stable even after exclusion of patients with concomitant hepatobiliary disease in a sensitivity analysis.

Despite these limitations, our data show that patients with alcohol consumption and malignant biliary strictures are at risk for enterococcal PLA. Enterococcal PLA, particularly PLA by *E. faecium* and other non-faecalis enterococci, indicates a higher risk of mortality, underscoring the need for close clinical monitoring and individualized treatment protocols in these patients.

## Abbreviations

PLA: Pyogenic liver abscess
MRSA: Methicillin-resistant Staphylococcus aureus
MRSE: Methicillin-resistant Staphylococcus epidermidis
VRE: Vancomycin-resistant enterococci
ESBL: Extended-spectrum beta-lactamase
CR: Carbapenem-resistant
